# Efficient Staining-Invariant Nuclei Segmentation Approach Using Self-Supervised Deep Contrastive Network

**DOI:** 10.3390/diagnostics12123024

**Published:** 2022-12-02

**Authors:** Mohamed Abdel-Nasser, Vivek Kumar Singh, Ehab Mahmoud Mohamed

**Affiliations:** 1 Department of Electrical Engineering, Aswan University, Aswan 81542, Egypt; 2 Computer Engineering and Mathematics Department, University Rovira i Virgili, 43007 Tarragona, Spain; 3 Department of Electrical Engineering, College of Engineering, Wadi Alddwasir, Prince Sattam Bin Abdulaziz University, Wadi Alddwasir 11991, Saudi Arabia

**Keywords:** whole slide imaging, hematoxylin and eosin (H&E), stain color normalization, nuclei segmentation, deep learning

## Abstract

Existing nuclei segmentation methods face challenges with hematoxylin and eosin (H&E) whole slide imaging (WSI) due to the variations in staining methods and nuclei shapes and sizes. Most existing approaches require a stain normalization step that may cause losing source information and fail to handle the inter-scanner feature instability problem. To mitigate these issues, this article proposes an efficient staining-invariant nuclei segmentation method based on self-supervised contrastive learning and an effective weighted hybrid dilated convolution (WHDC) block. In particular, we propose a staining-invariant encoder (SIE) that includes convolution and transformers blocks. We also propose the WHDC block allowing the network to learn multi-scale nuclei-relevant features to handle the variation in the sizes and shapes of nuclei. The SIE network is trained on five unlabeled WSIs datasets using self-supervised contrastive learning and then used as a backbone for the downstream nuclei segmentation network. Our method outperforms existing approaches in challenging multiple WSI datasets without stain color normalization.

## 1. Introduction

The digital pathology revolution began using a whole slide imaging (WSI) scanner to digitize glass slides. Digital pathology has been used in various applications, including case diagnosis and management, education for all clinical and patient cases, and forensic pathology. However, pathologists devote significant efforts to manual WSI image analysis (i.e., visual assessment of WSIs), particularly for tasks such as nucleus cell segmentation and counting [1].

In the literature, many computer analysis methods have been developed to analyze histopathology images [2,3,4,5]. Kleczek et al. [6] combined statistical analysis, color thresholding, and binary morphology to segment histopathological images of skin tissues. Kleczek et al. [7] proposed an automated method for epidermis segmentation in histopathological images of human skin. They incorporated the domain-specific details of morphometric and biochemical characteristics of skin tissue regions in histopathology images.

In recent years, deep learning approaches have been used to analyze histopathology images for various diagnosis tasks [8,9], such as nuclei cell counting, cancer metastasis detection, and forensic pathology applications such as determining the cause of death after trauma and poisoning. The automatic segmentation of nuclei in WSI images has been studied extensively. In [10], a five-step segmentation approach for nuclei cells or nanoparticles was proposed. The five steps were (1) automatic gradient image formation, (2) automatic threshold selection, (3) manual calibration of the threshold selection method for each cell or nanoparticle image, (4) manual determination of the segmentation cases for each specific cell or nanoparticle image type, and (5) automatic quantification by iterative morphological erosion. In [11], a selective-edge-enhancement-based nuclei segmentation method (SEENS) was proposed. In SEENS, a selective search algorithm was integrated with mathematical operators to segment cervical WSI images into small regions of interest while automatically evading duplicated segmentation and removing non-nuclei regions. An edge enhancement method based on the canny operator and mathematical morphology was used to extract edge information to enhance the nucleus edge.

In WSI image analysis, deep learning-based techniques, notably nucleus segmentation, are gaining popularity. In [12], various deep learning-based techniques were reviewed and assessed for breast tumor cell nuclei segmentation, including U-Net, Mask R-CNN, and GB U-Net. GB U-Net performed better in segmenting cell nuclei with an aggregated Jaccard index (AJI) score of 53%. Cui et al. [13] introduced an end-to-end deep learning network for nuclei segmentation that uses a nuclei boundary model to predict the inner nuclear instance, nuclear contour, and background in WSI images simultaneously. To improve and stabilize the inner nuclei and contour prediction, the authors used a weighted loss function based on the relative position of pixels inside the WSI image. They achieved an F1-score of 85.40% using the MoNuSeg dataset. Xie et al. [14] proposed the DIMAN method, a deep interval-marker-aware network, for nuclei segmentation. They integrated the convolutional neural networks with the marker-controlled watershed to delineate the foreground, marker, and interval of nuclei. DIMAN achieved an AJI score of 56.64% with the MoNuSeg dataset. Zhou et al. [15] introduced the U-Net++ model that combined UNets of various depths and restructured skip connections. They also used an architecture pruning approach to speed up inference while maintaining performance. On the MoNuSeg dataset, UNet++ had an F1-score of 88.17%.

Ilyas et al. [16] proposed a tissue-specific feature distillation network (TSFD-Net) trained with a combinational loss function to extract tissue-specific features from WSI images to produce better nuclei segmentation and classification. TSFD-Net was based on the fact that morphological features such as appearance, shape, and texture of nuclei in a tissue vary greatly depending upon the tissue type. With the PanNuke dataset, TSFD-Net obtained mean and binary panoptic quality of 50.4% and 63.77%, respectively. In an attempt to segment overlapped and clustered nuclei, Ref. [17] proposed the DenseRes-Unet model by integrating dense blocks in the last layers of the encoder block of U-Net, as well as distance map and binary threshold techniques to intensify the nuclei interior and contour information in WSI images. Rączkowski et al. [18] recommended an active (ARA) image classification method using Bayesian CNN that classifies colorectal cancer tissue. The authors designed a network that measures the uncertainty of the given test samples. This approach helped to identify the misclassified training samples and could improve the model performance. Hassan et al. [19] suggested a clustering-based stain selection technique. They trained a set of independent deep-learning models on several stain templates. The authors combined the segmentation masks of the individual models using an aggregation function based on the Choquet integral. Recently, self-supervised learning attained great success in analyzing histopathology images, where the trained models can extract rich features from the unlabeled data and later could be used to improve the downstream nuclei segmentation or classification performance [20,21,22].

**Figure 1:**
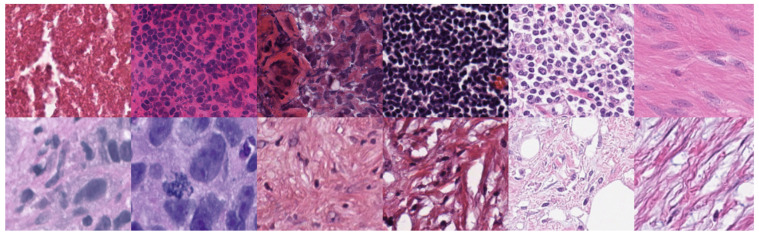
Examples of WSIs having various stains. The present nuclei have different shapes and sizes.

Existing nuclei cell segmentation approaches require a stain color normalization step to reduce color variations in WSIs due to various stains used in laboratories and stain manufacturing processes across vendors. Selecting a proper staining normalization method is crucial for the nuclei segmentation methods. However, staining normalization methods have some limitations, such as (1) they cannot handle the problem of inter-scanner feature instability; (2) they modify the color of WSIs, which may yield a loss in the source domain information—they do not preserve the source intensity variation (notably, source color variation can help with WSI analysis, as it can reveal crucial differences in the tissue’s underlying biochemical composition); and (3) they may produce inconsistent color normalization results when the number of stains increases (resulting WSIs deviate from the target staining template). In addition, most nuclei cell segmentation methods still face challenges due to the variations in nuclei shapes and sizes and overlapping and clumped cell nuclei. Figure 1 shows WSI images obtained from different organs and multiple laboratories. As one can see, there is a big variation in the stain color, nuclei shapes and sizes, and the presence of overlapping and clumped nuclei. Such differences could greatly reduce the accuracy of automated nucleus cell segmentation systems.

Unlike most existing nuclei segmentation approaches that require a staining normalization algorithm, we propose an efficient staining-invariant nuclei segmentation method based on self-supervised contrastive learning and an effective weighted hybrid dilated convolution (WHDC) block. Specifically, we propose a staining-invariant encoder (SIE) that includes convolution (Conv) and transformers blocks [23], where Conv blocks help extract low-level nuclei features, and transformer blocks model their long-range relationships. We also propose a WHDC block to enable the network to learn multi-scale features to handle the variation in the size and shapes of nuclei. SIE is trained using the SimCLR contrastive learning framework [24] in a self-supervised manner that learns latent staining-invariant representations of WSIs without any labeled data. The staining invariant encoder is used as a backbone, followed by a supervised fine-tuning strategy for the nuclei segmentation task. The key contributions of this article are as follows:

- Proposing an efficient nuclei segmentation method for hematoxylin and eosin (H&E) WSI images using a deep staining-invariant self-supervised contrastive network. This method eliminates the need for a stain normalization step;
- Proposing an effective weighted hybrid dilated convolutional (WHDC) block that helps extract multi-scale nuclei-relevant representations;
- Achieving accurate nuclei segmentation on unseen single-organ and multi-organ datasets collected from different laboratories without employing stain color normalization or fine-tuning that demonstrate the proposed method’s generalization capabilities.

It should be noted that the proposed method is not limited and could be employed for other applications such as nanoparticle segmentation.

## 2. Proposed Method

**Figure 2:**
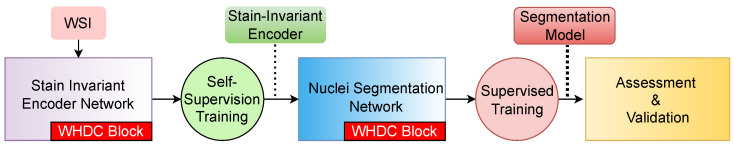
Illustration of the proposed method pipeline.

Figure 2 depicts the pipeline of the proposed method. A staining-invariant encoder is trained using self-supervised contrastive learning (Section 2.1). The encoder network includes the proposed WHDC block to handle the variation in the size and shapes of nuclei (Section 2.2). This encoder is a backbone for the downstream nuclei segmentation network trained using a supervised fine-tuning strategy (Section 2.3). Details are given below.

### 2.1. Staining-Invariant Encoder

**Figure 3:**
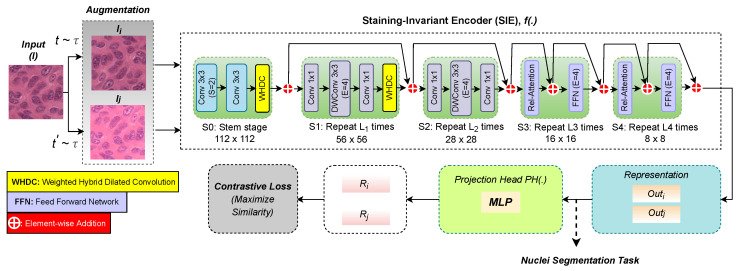
The architecture of proposed staining-invariant encoder (SIE) based on self-supervised contrastive learning.

Figure 3 presents the overview of the proposed staining-invariant encoder (SIE) network. The main components of SIE are convolution blocks, transformer blocks, and the proposed WHDC block. In particular, SIE is based on a convolutional-transformer neural network so-called CoAtNet [23]. SIE is trained using a self-supervised contrastive learning approach [24] that does not demand any labeled dataset prepared by pathologists. In other words, the training of SIE is completely based on the self-supervised learning technique, in which the model learns patterns by itself by extracting stain-invariant nuclei-relevant features.

As shown in Figure 3 (left), SIE extracts feature representations from pairs of augmented WSI images. We apply data augmentation techniques to construct pairs of WSI images, $I_{i}$, and $I_{j}$, to learn visual feature similarities between them. Specifically, we apply the following data augmentation techniques: flipping, rotation with 90 degrees, blurring, random brightness contrast with a probability of 0.2, and color jitter. SIE learns visual features through a contrastive loss function by increasing the agreement among different augmented views of the same WSI image patch example.

**Figure 4:**
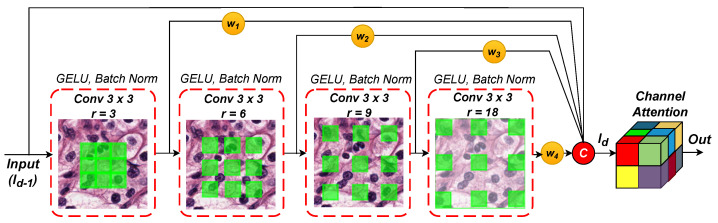
Illustration of the WHDC block.

The top branch shown in Figure 3 represents the backbone feature extractor (i.e., *f*), which is based on CoAtNet [23] that includes convolution (Conv) and transformer blocks [23]. SIE has five stages (S0, S1, S2, S3, and S4). The first three stages rely on convolution blocks, whereas the last two adopt transformer blocks. Stage S0 applies a down-sampling operation with a factor of 2 to reduce the patch image spatial size. The first stage has two standard convolution layers with a kernel size of 3×3, allowing for extraction of nuclei-relevant features (e.g., shape, texture, and intensity) from WSI patches. As there is a wide variety of nuclei sizes, we use the WHDC block to encourage the model to learn multi-scale nuclei-relevant features. WHDC employs dilation rates of 3, 6, 9, and 18, where the small receptive fields capture the fine details of nuclei, and the larger receptive field provides contextual information (Figure 4). WHDC has a self-weighing mechanism that encourages the network to promote nuclei-relevant features.

The second stage, S1, contains an MBConv block (i.e., inverted residual block) [23]. The MBConv substitutes the conventional convolution with the depth-wise separable convolutions. WHDC is also added at the end of S1 with the same parameter setting used in S0. The architecture of S2 is identical to S1 but without WHDC. Stages S3 and S4 contain a transformer block that encourages the SIE network to establish long-range dependencies while avoiding overfitting using a 2D relation attention and feed-forward network (i.e., FFN) and a self-attention module. Stages S3 and S4 apply a max-pooling operation with a stride of 2 on the self-attention module’s constituents. The final size of the bottleneck of S4 is 8×8 (at the arrow connecting the top and bottom branches in Figure 3). The output of the top branch of the SIE network, *f*, can be formulated as follows:

(1) $${Out}_{i}=f\left({\tilde{I}}_{i}\right)$$

(2) $${Out}_{j}=f\left({\tilde{I}}_{j}\right)$$

where $Out_{i}$, and $Out_{j}\in R^{d}$ stand for the output representations for the augmented image pairs $I_{i}$ and $I_{j}$, respectively.

The projection head PH(.) projects the generated representations $Out_{i}$ and $Out_{j}$ into a 128-dimensional feature space using only a single-layer MLP (multi-layer perceptron). MLP computes the representation $R_{i}$ for $Out_{i}$ and a representation $R_{j}$ for $Out_{j}$ as follows:

(3) $$R_{i}=PH\left({\mathrm{Out}}_{i}\right)=W^{\left(2\right)}\sigma \left(W^{\left(1\right)}{\mathrm{PH}}_{i}\right)$$

(4) $$R_{j}=PH\left({\mathrm{Out}}_{j}\right)=W^{\left(2\right)}\sigma \left(W^{\left(1\right)}{\mathrm{PH}}_{j}\right)$$

where *W* stands for the weights of MLP, and σ is a non-linear rectified linear unit (ReLU) function.

SIE is built using a self-supervised contrastive learning approach [24], which does not require any labeled dataset. The contrastive loss can be formulated as

(5) $$L_{FINAL}=\frac{1}{2N}\sum_{k=1}^{N}\left[\ell \left(2k-1,2k\right)+\ell \left(2k,2k-1\right)\right]$$

where *N* stands for the mini-batch WSI patch images and contrastive prediction result to 2N data points computed through pairs of data-augmented patch samples. The *ℓ* can be computed as

(6) $$\ell_{i,j}=-\log\frac{\exp\left(\mathrm{CM}\left(R_{i},R_{j}\right)/\tau \right)}{\sum_{k=1}^{2N}1_{\left[k\neq i\right]}\exp\left(\mathrm{CM}\left(R_{i},R_{k}\right)/\tau \right)}$$

where τ stands for a temperature parameter set to 0.07 in our experiments; $1_{\left[k\neq i\right]}\in \left\{0,1\right\}$ corresponds to an indicator function to measure if k≠i; CM is the cosine similarity function that can be defined as

(7) $$\mathrm{CM}\left(R_{i},R_{j}\right)=R_{i}^{T}R_{j}/∥R_{i}∥∥R_{j}∥$$

Contrastive loss decreases when projections from the same image are similar; otherwise, the error rate will increase.

### 2.2. Weighted Hybrid Dilated Convolution (WHDC) Block

Figure 4 presents the proposed WHDC block to widen the receptive fields of SIE with different dilation rates and encourage it to promote multi-scale nuclei features. WHDC consists of four cascaded convolutions layers, self-weighting, and channel attention mechanisms. It incorporates various spatial scales that provide fine pixel-level details and global contextual information without losing resolution size. In this study, we use dilation rates of 3, 6, 9, and 18. As shown in Figure 4, WHDC has four convolutional layers with a kernel size of 3×3 followed by batch normalization and a non-linear GELU activation function connected in a cascaded manner. The WHDC block allows for utilizing the extracted features from the previous layer with specific dilation rates and feeds them into the next convolutional layer. In WHDC, a convolutional layer of depth *d* and dilation rate with *r* can be expressed as follows:

(8) $${Dilated-Conv}_{d}^{r}:I_{d-1}⟶I_{d}^{r}$$

where the input of each dilated convolution has a size of *h*, *w*, and *c* (i.e., height, width, and number of channels, respectively), and $I_{d-1}\in R^{h^{\prime }\times w^{\prime }\times c^{\prime }}$. WHDC produces feature maps of size $I_{d-1}\in R^{h\times w\times c}$.

As shown in Figure 4, the proposed WHDC block has a self-attention mechanism to promote the nuclei-relevant features while ignoring other irrelevant features. It should be noted that the four weights of the self-attention mechanism, $w_{1}$, $w_{2}$, $w_{3}$, and $w_{4}$, are derived dynamically based on a Sigmoid activation function. The weighted features ($w_{1}*I^{r=3}$, $w_{2}*I^{r=6},w_{3}*I^{r=9}$, and $w_{4}*I^{r=18}$) and original input $I_{d-1}$ are concatenated and fed into a channel attention mechanism (CAM) [25] to advance channel interdependencies. CAM converts the concatenated nuclei feature maps output to a single vector through a global pooling layer named squeeze operation. Subsequently, CAM uses two fully connected (FC) layers with a channel reduction ratio of 16. For further details about the architecture of CAM and its FCs, the readers are recommended to see [25]. The weights of each channel are multiplied with the original input vector to boost nuclei-relevant features (i.e., excitation operation) automatically.

### 2.3. Nuclei Segmentation Network

**Figure 5:**
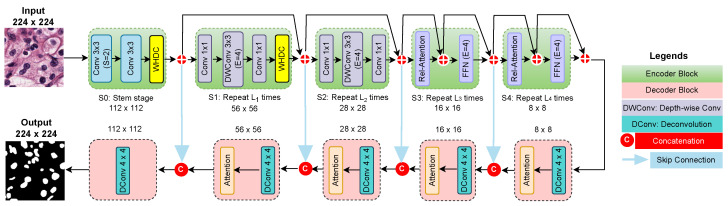
The framework of the proposed nuclei segmentation network.

Figure 5 presents the proposed nuclei segmentation network for WSI images. It includes an encoder and decoder network. SIE trained with contrastive learning (Section 2.1) is used as an encoder. The encoder’s bottleneck features (8×8 feature maps) are fed into the decoder network (i.e., the network at the bottom of Figure 5). The decoder consists of five layers. Each decoder layer utilizes a Conv-transpose layer with a kernel size of 4×4 and stride of 2. In this study, we adopt the attention mechanism [26] to initial four decoder layers that ignore irrelevant artifacts generated during feature reconstruction and concentrate only on nuclei-relevant features that lead to reducing the false positives. Except for the last layer, each decoder layer has batch normalization and ReLU activation functions. Skip connections between encoder and decoder networks are employed to narrow the semantics gaps in feature reconstruction. We use a threshold value of 0.5 to generate the final binary segmentation masks.

We fuse two loss functions to train the segmentation network—binary cross-entropy (BCE) and Dice losses. This combined loss function could minimize the error and address the pixel imbalance issue between the nuclei and background areas. The BCE loss is defined as

(9) $$\begin{array}{cc} L^{\mathrm{BCE}}\left(y,t\right)= & -(y\cdot log(t)+(1-y)\cdot log(1-t)) \end{array}$$

where *y* and *t* stand for the ground truth mask and mask generated by the proposed segmentation network. The Dice loss can be expressed as

(10) $$L^{\mathrm{Dice}}\left(y,t\right)=1-Dice\left(y,t\right)=1-\frac{2|y|.|t|}{{\left|y\right|}^{2}+{\left|t\right|}^{2}},$$

The overall segmentation loss (OSL) is the weighted sum of $L^{\mathrm{BCE}}$ and $L^{\mathrm{Dice}}$:

(11) $$L^{\mathrm{OSL}}\left(y,t\right)=\gamma L^{\mathrm{BCE}}\left(y,t\right)+\left(1-\gamma \right)L^{\mathrm{Dice}}\left(y,t\right)$$

where γ is a weighting factor experimentally set to 0.4.

## 3. Results and Discussion

### 3.1. Datasets

In this study, we leveraged a total of eight publicly available datasets, including Lizard [27], MHIST [28], BreCaHD [29], SPIE-BreastPathQ [30], Colorectal NCT-CRC-HE [31], MoNuSeg [32], CryoNuSeg [33], and BNS [34].

To build the staining-invariant encoder (i.e., SIE) based on contrastive learning, we used Lizard, MHIST, BreCaHD, SPIE-BreastPathQ, and Colorectal NCT-CRC. The Lizard dataset has a half-million labeled nuclei in H&E stained colon tissue with 20× objective magnification. The entire set involves 291 images with an average resolution of 1016×917. The MHIST has 3152 H&E-stained colorectal polyp images with 224×224 pixels. BreCaHAD contains 162 breast cancer WSIs with a resolution of 1360×1024. SPIE-BreastPathQ has 96 H&E WSI scans acquired from 55 patients with residual invasive breast cancer. This dataset has a patch size of 512×512 and comprises training, validation, and test sets of 2394, 185, and 1119 images, respectively. Colorectal NCT-CRC-HE includes 100 thousand non-overlapping patches derived from 86 H&E stained human cancer 54 tissue slides of colorectal cancer and normal tissue. The size of the patches is 224×224.

To construct and evaluate the nuclei segmentation model, we used the MoNuSeg, CryoNuSeg, and BNS datasets. MoNuSeg is a multi-center multi-organ dataset containing 30 WSIs with a resolution of 1000×1000. It has a total of 21 thousand manually annotated nuclei. MoNuSeg involves WSI of seven organs—breast, kidney, colon, stomach, prostate, liver, and bladder. A total of 23 WSIs are used for training and 7 WSIs (i.e., one WSI per organ) for a fair assessment.

To train the segmentation model, we resized the original WSIs to the size of 1024×1024. Then, we applied non-overlapping cropping with patch size 512×512. To increase the number of training samples, for each non-overlapping patch, we applied random crops, generating 200 samples of patch size 256×256. In total, we generated 18,400 (23×4×200) training samples. BNS has 33 WSIs with a resolution of 512×512 for breast cancer (2754 labeled nuclei). CryoNuSeg has WSIs of 10 human organs—adrenal gland, larynx, lymph node, mediastinum, pancreas, pleura, skin, testis, thymus, and thyroid gland. It has 30 WSIs with a resolution of 512×512. It is worth noting that BNS and CryoNuSeg datasets are independently used for evaluating the proposed model, meaning they are not used for training or fine-tuning the segmentation model.

### 3.2. Implementation Details

The training process is two-fold—training the staining-invariant encoder network (i.e., SIE) based on contrastive learning (Section 2.1) and training the nuclei segmentation network (Section 2.3). We applied data augmentation techniques, including flipping, 90-degree rotation, blurring, random brightness contrast with a probability of 0.2, and color jitter. The input image size was 224×224. The SIE network is trained using an SGD optimizer with an initial learning rate of 0.001. The number of epochs is 50, with a mini-batch size of 2. For training the nuclei segmentation network, the number of epochs is set to 100 with a mini-batch size of 4. ADAM optimizer is used with $\beta_{1}$ = 0.5 and $\beta_{2}$ = 0.999 and a learning rate of 0.0002. In our experiments, all hyperparameters are manually tuned. We used the same hyperparameter settings for the proposed model and state-of-the-art models presented in this study. The proposed method is implemented on PyTorch 1.7.0, CUDA 11.2 on Intel Core-i9 with 32 GB RAM, and GeForce RTX 3090 GPU with 24 GB memory.

### 3.3. Evaluation Metrics

To assess the performance of segmentation methods, we used the dice coefficient (Dice), aggregated Jaccard index (AJI) [32], precision, and recall. These metrics can be expressed as follows:

(12) $$Dice=\frac{2\cdot TP}{2\cdot TP+FP+FN},$$

(13) $$Precision=\frac{TP}{\left(TP+FP\right)},$$

(14) $$Recall=\frac{TP}{\left(TP+FN\right)},$$

(15) $$\mathrm{AJI}=\frac{\sum_{i=1}^{L}\left|GT_{i}\cap N\psi_{j}^{*}\left(i\right)\right|}{\sum_{i=1}^{K}\left|GT_{i}\cup N\psi_{j}^{*}\left(i\right)\right|+\sum_{K\in \mathrm{LIP}}N\psi_{k}}$$

In these expressions, TP, FP, FN, and TN rates stand for true positive, false positive, false negative, and true negative, respectively; $GT_{t}$, $N\psi_{k}$, and $N\vartheta_{j}^{*}\left(i\right)$ stand for the *i*th ground-truth mask of nuclei pixels, the predicted nuclei segmentation mask, and the connected component from the predicted mask that maximizes the Jaccard index, respectively; LIP stands for the list of indices of pixels that do not belong to any element in GT.

### 3.4. Ablation Study

Here, we conducted a thorough ablation study to demonstrate the efficacy of the proposed model’s specific components, where we investigated the effect of various configurations of the proposed segmentation model and the nuclei segmentation results of different loss functions.

#### 3.4.1. Analysis of Various Configurations

Table 1 presents the ablation study of different configurations of the proposed nuclei segmentation network—baseline (i.e., BL), baseline+WHDC, SIE without WHDC, SIE without contrastive learning (CL) approach (i.e., proposed w/o CL), and the proposed SIE network (i.e., network with all components). In this table, we present the mean and the standard deviation (SD) of all evaluation metrics across all the test samples.

We assessed the performance of the BL network that entirely relies on the encoder and decoder network without adopting the WHDC block or CL. BL obtained Dice and AJI scores of 83.32% and 66.69%, respectively. We added the WHDC block with the BL network, which boosted the Dice and AJI scores by 1.1% to 2%. This configuration allows for the extraction of spatial nuclei-relevant features comprising shape, texture, and intensity while avoiding irrelevant ones. In turn, feature reconstruction is an important step in creating segmentation maps. We leverage the spatial attention mechanism in the decoder to prevent losing the semantic correlations. BL+WHDC with spatial attention mechanism in the encoder (proposed w/o CL) improved the segmentation performance by 2% to 3% in Dice, AJI, precision, and recall scores when compared to BL. The proposed model incorporated the pre-trained SIE model trained with contrastive learning and WHDC. Pre-trained SIE provided staining-invariant nuclei-relevant features, while the proposed WHDC block helped generate multi-scale nuclei features. This led to a significant gain of 5% to 6% in all evaluation metrics. We also observed the proposed model generated fewer false positive pixels leading to a lower SD of 5% in Dice and IoU scores against the BL model.

**Figure 6:**
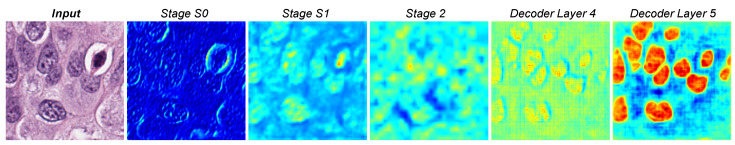
Heatmaps from encoding and decoding layers of the proposed segmentation model.

Figure 6 presents heatmaps from the encoding and decoding layers of the proposed segmentation model. As one can see, stage S0 extracts the spatial nuclei features such as shape, texture, and intensity. Stages S1 and S2 emphasize nuclei features with finer details, due to the proposed WHDC block, which encourages the network to learn multi-scale nuclei-relevant feature representation. Due to low spatial resolution (16×16 and 8×8), we do not show the feature maps of stages S3, S4, and the early decoder layer output. In addition, the decoder layers 4 and 5 also show that the segmentation model correctly identified the nuclei region with sharp boundaries (highlighted in red) while ignoring the background.

#### 3.4.2. Analysis of the Loss Function

Table 2 presents the effect of different loss functions (i.e., $L^{\mathrm{BCE}}$, $L^{\mathrm{Dice}}$, and $L^{\mathrm{BCE}}$ + $L^{\mathrm{Dice}}$) on the efficiency of the proposed segmentation network evaluated with the MoNuSeg dataset. We used two loss functions consisting of $L^{\mathrm{BCE}}$ and $L^{\mathrm{Dice}}$ losses. We initialized our training by only using the $L^{\mathrm{BCE}}$ loss that provides a Dice score of 84.76% and an AJI score of 69.2%. The $L^{\mathrm{Dice}}$ loss was employed to focus more on dense pixel prediction by generating fewer false positives; $L^{\mathrm{Dice}}$ achieves the 83.71% and 68.97% Dice and AJI scores, respectively. Both $L^{\mathrm{BCE}}$ and $L^{\mathrm{Dice}}$ performed well, and thus we combined them to achieve better results with lower false-positive rates. We set the weighting factor γ to 0.4 (Equation (11)). The combined loss functions reduce SD in the range of 3−6% for all the evaluated metrics against $L^{\mathrm{BCE}}$. The ablation study confirmed that each loss function reasonably contributed to the final nuclei segmentation (4% improvement in Dice and AJI scores).

### 3.5. Comparison with Existing Methods

Table 3 compares the proposed method with 12 state-of-the-art networks on the MoNuSeg dataset. We trained 5 networks of these 12 networks from scratch, meaning the 5 networks were completely trained without utilizing any pre-trained ImageNet weights. The five networks trained from scratch using the same hyperparameters used for training the proposed model are U-Net, fully convolutional network (FCN), DeepLabv3+, Attention U-Net [26], and U-Net++ [35] with the same hyperparameter settings. The findings of the other seven methods are taken from recently published nuclei segmentation studies. As tabulated in Table 3, the proposed model outperformed state-of-the-art methods by a significant margin and achieved a SD ranging from 6 to 9% in all evaluation metrics, which is much lower than the other compared methods. It achieves Dice, AJI, precision, and recall scores of 88.64%, 73.14%, 88.2%, and 89.1%, respectively. The U-Net, DeepLabv3+, and FCN obtained Dice scores of 77.94%, 76.59%, and 76.36% respectively, which are 10% lower than the proposed method. Both Attention U-Net [26] and U-Net++ [35] obtain an average Dice of 79.5%. The proposed model attains 2% and 1% improvements in the Dice and AJI scores, respectively, which are higher than the second-best method cGAN [36]. The cGAN-based approach generated synthetic nuclei images and combined them with original training data to segment the nuclei areas. The RIC-UNet [37], DIST [38], MedT [39], Chanchal et al. [40], and BiO-Net [41] achieve Dice scores lower than 83%. Although MSAL-Net [42] used a multi-scale attention learning network with dense dilated convolution, it provides a Dice score of 83.9%, which is 4.5% lower than our method.

**Figure 7:**
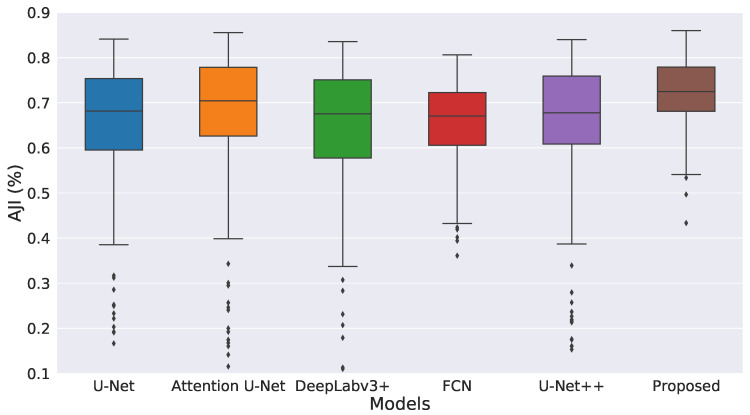
Boxplot of AJI scores of the proposed nuclei segmentation method on MoNuSeg dataset.

Figure 7 presents statistics of AJI score of the proposed method, U-Net, Attention U-Net [26], DeepLabv3+, FCN, and U-Net++ [35]. Our model achieves the highest mean and median scores and lowest standard deviation among other compared methods. It only has three outliers, whereas other methods have many outliers with large standard deviations.

**Figure 8:**
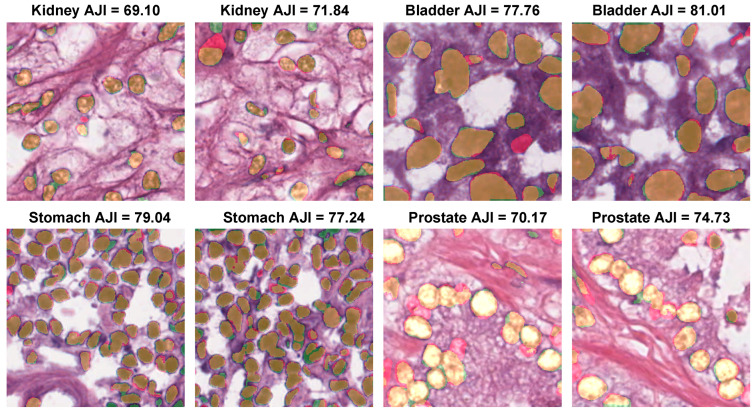
Nuclei segmentation by the proposed method in WSIs of four organs. The color maps are displayed as follows: true positives (TP: orange), false positives (FP: green), false negatives (FN: red), and true negatives (TN: background).

Figure 8 presents the nuclei segmentation results of the proposed method with WSI images collected from different laboratories for kidney, bladder, stomach, and prostate organs. We provided the color maps to easily interpret the segmentation results compared to the ground truth. With the MoNuSeg dataset, the proposed method could precisely capture the different nuclei sizes and segment the nuclei boundaries (orange color) with very few false positives (green color).

**Figure 9:**
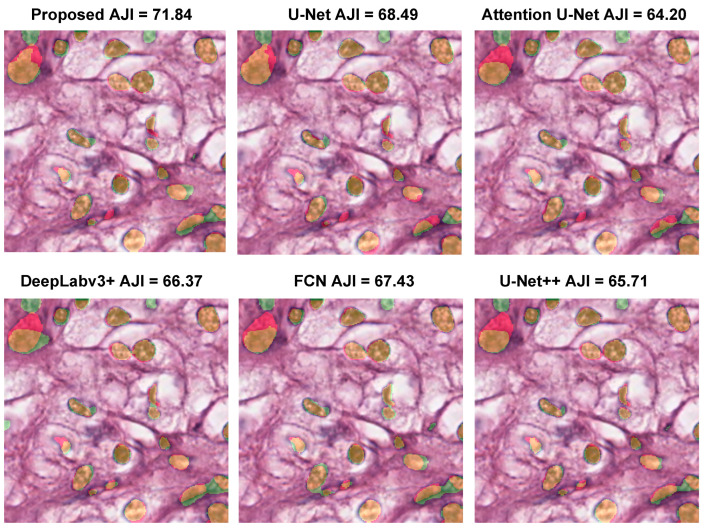
Comparison of the proposed model with five existing methods on the MoNuSeg dataset. The color maps are displayed as follows: true positives (TP: orange), false positives (FP: green), false negatives (FN: red), and true negatives (TN: background).

Figure 9 shows qualitative segmentation results of the proposed method compared to U-Net, Attention U-Net [26], DeepLabv3+, FCN, and U-Net++ [35]. The AJI scores demonstrated the quantitative improvement produced by the proposed segmentation method (71.84%) compared to other approaches. The proposed model produces excellent segmentation results with fewer false positives of small nuclei, whereas other methods do not completely segment many nuclei. In addition, the Wilcoxon signed-rank test demonstrated that the results of the proposed model and second-best U-Net++ on the MoNuSeg dataset were statistically significant (*p*-value <0.001).

### 3.6. Evaluating the Proposed Method on Other Datasets

Herein, we evaluate the effectiveness of the proposed model trained on the MoNuSeg dataset using the CryoNuSeg and BNS datasets without retraining or fine-tuning (the complete dataset is used as the test set). We independently trained the U-Net, Attention U-Net [26], DeepLabv3+, FCN, and U-Net++ [35] on the CryoNuSeg and BNS datasets from scratch. As tabulated in Table 4, the proposed method outperformed other segmentation approaches with Dice, AJI, precision, and recall scores of 86.53%, 64.7%, 85.48%, and 87.62%, respectively. In the case of the proposed method, the estimation errors (i.e., SD) of the Dice and AJI scores are 1% lower than for U-Net. Hassan et al. [19] achieved the second-best results, which was 1% less than our method. As tabulated in Table 5, the proposed method outperformed the other methods. DeepLabv3+ obtained poor segmentation results with limited samples. The U-Net++ achieved the second-best results with an 83.39% Dice score and a 62.72% AJI. As one can see, the estimation errors of the proposed method in terms of the Dice and AJI scores are 1% lower than for U-Net++. Although CryoNuSeg and BNS datasets were entirely unseen by the proposed segmentation model, they achieved the best results, thanks to the robust multi-scale nuclei-relevant staining-invariant feature representations learned by the model.

**Figure 10:**
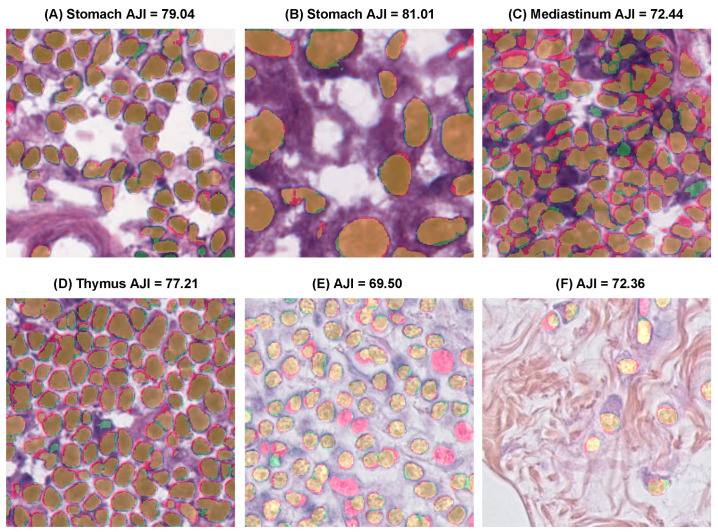
The segmentation results of the proposed method on (**A**,**B**) MoNuSeg, (**C**,**D**) CryoNuSeg, and (**E**,**F**) BNS. The color maps are displayed as follows: true positives (TP: orange), false positives (FP: green), false negatives (FN: red), and true negatives (TN: background).

Figure 10 shows the segmentation results of the proposed method on the MoNuSeg, CryoNuSeg, and BNS datasets. These WSI were collected from different organs in laboratories employing various stain colors. However, the proposed method could accurately segment nuclei. These findings proved the generalization abilities of the proposed method, and it could segment nuclei in WSI images without employing stain color normalization or fine-tuning the model.

### 3.7. Discussion and Limitations

Although the existing methods achieved acceptable results, they required a stain color normalization, which can differ from one dataset to another and may yield a loss in the source domain information (other limitations stated in Section 1). In addition, existing methods could not distinctly delineate the nuclei boundary, leading to several false positives. Adopting a self-supervising contrastive learning approach and the proposed WHDC block to build a stain-invariant encoder encouraged the segmentation model to concentrate on segmenting and separating the nuclei boundaries. It is evident that the proposed model showed great potential and provided an efficient solution to segment nuclei in WSIs of different stains and multiple organs and surpasses the existing deep learning approaches by a significant margin.

One of the limitations of the proposed method is that it produces poor segmentation results in the case of overlapped and clumped nuclei.

## 4. Conclusions and Future Work

This paper proposed an effective staining-invariant nuclei segmentation method based on a self-supervised contrastive learning approach. In particular, we introduced a staining-invariant method that does not use color normalization before processing H&E WSI. The proposed staining invariant encoder (SIE) leveraged the convolution, WHDC, and transformer blocks in a self-supervised training setting that facilitates learning better nuclei feature representation. A trained SIE model was used as the backbone for the downstream nuclei segmentation task. We used eight WSI datasets, including five datasets for training the self-supervised SIE network, and the remaining three were applied to assess the effectiveness of the proposed nuclei segmentation model. The proposed method achieved state-of-the-art AJI scores of 73.14%, 64.7%, and 65.20% with MoNuSeg, CryoNuSeg, and BNS datasets, respectively. Our analysis showed that the proposed method achieved accurate nuclei segmentation on a completely unseen independent dataset, due to the robust multi-scale nuclei-relevant staining-invariant feature representations learned by the SIE model. It also demonstrated the generalization capabilities of the proposed method on multiple datasets, and it could segment nuclei in WSI images without employing stain color normalization or fine-tuning the model. It is worth noting that the proposed staining-invariant method is not limited and can be applied to other applications, such as nanoparticle segmentation, which will be the focus of future work.

## Figures and Tables

**Table 1:** Ablation study on MoNuSeg. The best results are highlighted in bold.

Model	Dice (%) ↑	AJI (%) ↑	Precision (%) ↑	Recall (%) ↑
BL	83.32±11.80	66.69±13.17	82.11±15.12	84.58±18.03
BL + WHDC	84.46±10.52	68.56±12.73	83.97±13.93	84.95±16.87
Proposed w/o CL	85.82±9.98	69.11±11.41	85.19±11.84	86.46±12.76
**Proposed**	88.64±6.20	73.14±8.19	88.2±7.38	89.1±8.09

**Table 2:** Ablation study of the loss functions on MoNuSeg. The best results are highlighted in bold.

Loss Function	Dice (%) ↑	AJI (%) ↑	Precision (%) ↑	Recall (%) ↑
$L^{\mathrm{BCE}}$	84.76±11.46	69.2±13.67	83.47±12.94	86.03±11.88
$L^{\mathrm{Dice}}$	83.71±11.81	68.97±14.93	82.01±13.02	85.48±12.20
$L^{\mathrm{BCE}}$ + $L^{\mathrm{Dice}}$	88.64±6.20	73.14±8.19	88.2±7.38	89.1±8.09

**Table 3:** Comparing the proposed method with 12 existing methods on the MoNuSeg dataset. The − symbol represents the missing value that was not reported in the study. The best results are highlighted in bold.

Model	Dice (%) ↑	AJI (%) ↑	Precision (%) ↑	Recall (%) ↑
U-Net	77.94±17.71	64.01±17.23	76.69±20.65	79.24±23.96
Attention U-Net	79.52±19.44	65.22±18.82	78.36±19.89	80.73±24.80
DeepLabv3+	76.59±25.75	60.86±22.43	74.28±24.81	79.05±29.47
FCN	76.36±9.60	65.01±10.19	73.86±12.34	79.05±14.59
U-Net++	79.57±18.80	63.92±18.01	78.02±19.81	81.19±24.85
RIC-UNet [37]	82.78	56.35	−	−
DIST [38]	78.63	55.98	−	−
Chanchal et al. [40]	80.65	67.95	−	−
cGANs [36]	86.60	72.10		
MedT [39]	79.55	66.17	−	−
MSAL-Net [42]	83.9	70.6	82.1	85.3
BiO-Net [41]	82.5	70.4	−	−
**Proposed**	88.64±6.20	73.14±8.19	88.2±7.38	89.1±8.09

**Table 4:** Comparing the proposed method with existing approaches on the CryoNuSeg dataset. The best results are highlighted in bold.

Model	Dice (%) ↑	AJI (%) ↑	Precision (%) ↑	Recall (%) ↑
U-Net	81.01±9.15	59.62±11.13	78.33±8.42	83.89±6.97
Attention U-Net	81.87±8.70	60.58±10.48	79.81±7.77	84.06±7.63
DeepLabv3+	84.20±10.87	62.07±12.74	81.96±7.63	86.57±5.74
FCN	84.94±10.20	62.34±12.19	82.77±7.19	87.23±5.57
U-Net++	83.41±11.05	61.51±12.66	80.59±6.55	86.44±5.98
Hassan et al. [19]	85.55	64.51	85.02	86.16
**Proposed**	86.53±8.67	64.7±10.22	85.48±5.13	87.62±6.72

**Table 5:** Comparing the proposed method with existing approaches on the BNS dataset. The best results are highlighted in bold.

Model	Dice (%) ↑	AJI (%) ↑	Precision (%) ↑	Recall (%) ↑
U-Net	82.40±10.52	60.86±11.15	81.78±8.65	83.02±7.85
Attention U-Net	83.39±9.87	61.20±10.69	82.94±8.03	83.71±7.50
DeepLabv3+	78.44±14.58	55.29±17.82	77.53±11.48	79.38±10.83
FCN	81.38±11.53	59.63±12.94	80.50±9.11	82.28±8.46
U-Net++	83.89±7.47	62.72±9.07	85.24±8.33	87.92±7.39
**Proposed**	88.82±6.69	65.2±7.81	87.64±7.36	90.04±5.72

## Data Availability

The authors confirm that all datasets used in this study are publicly available and cited in the article.
